# The Effect of Processing Conditions on the Microstructure of Homopolymer High-Density Polyethylene Blends: A Multivariate Approach

**DOI:** 10.3390/polym16070870

**Published:** 2024-03-22

**Authors:** Fulvia Cravero, Nicola Cavallini, Rossella Arrigo, Francesco Savorani, Alberto Frache

**Affiliations:** 1 Department of Applied Science and Technology, Politecnico di Torino, Viale Teresa Michel 5, 15121 Alessandria, Italy; fulvia.cravero@polito.it (F.C.); alberto.frache@polito.it (A.F.); 2 Local INSTM Unit, 15121 Alessandria, Italy; 3 Department of Applied Science and Technology, Politecnico di Torino, Corso Duca degli Abruzzi 24, 10129 Torino, Italy; nicola.cavallini@polito.it (N.C.); francesco.savorani@polito.it (F.S.)

**Keywords:** HDPE blends, melt compounding, rheology, PCA, DoE

## Abstract

In this work, a multivariate approach was utilized for gaining some insights into the processing–structure–properties relationships in polyethylene-based blends. In particular, two high-density polyethylenes (HDPEs) with different molecular weights were melt-compounded using a twin-screw extruder, and the effects of the screw speed, processing temperature and composition on the microstructure of the blends were evaluated based on a Design of Experiment–multilinear regression (DoE-MLR) approach. The results of the thermal characterization, interpreted trough the MLR (multilinear regression) response surfaces, demonstrated that the composition of the blends and the screw rotation speed are the two most important parameters in determining the crystallinity of the materials. Furthermore, the rheological data were examined using a Principal Component Analysis (PCA) multivariate approach, highlighting also in this case the most prominent effect of the weight ratio of the two base polymers and the screw rotation speed.

## 1. Introduction

Polyethylene (PE) is one of the most commonly exploited and widespread thermoplastic polymers, owing to its low manufacturing costs, easy processability, good mechanical properties and very good chemical resistance [1,2,3]. The automotive industries, packaging, wires and plumbing represent the major users of this commodity polymer [1,3,4,5,6]. In fact, these markets require such a quantity of PE that by 2023, an annual global production of 157 million tons is estimated [2].

However, approximately 70% of PE is used as a blend [7]. Thus, considering the huge quantity of PE blends that are on the market and the strong dependence of the final properties on their inner microstructure, this topic has gained increasing attention in the scientific community over the years. In fact, several studies focused specifically on the miscibility of different kinds of PE blends, both in their solid and molten state. In particular, binary blends involving high-density (HDPE), low-density (LDPE) and linear low-density (LLDPE) polyethylenes have been studied [7,8,9,10,11,12,13,14]. Nonetheless, some disagreements between these studies still persist, specifically on the actual miscibility depending on the liquid or molten state of the blend and on the characterization methods used [7,8,9,15]. In addition, when addressing the effect of different factors on the final characteristics of the blends, the studies mainly focus on the effect of the macromolecular architecture (i.e., molecular weight, molecular weight distribution and branch length) of the considered polymers, while little attention has been paid to the influence of the processing parameters [7,8,9,11,12,14]. Furthermore, as reported by Zhao et al. [7], most of the miscibility studies consider temperatures that are much lower than those that are usually used during processing. In particular, to the best of the authors’ knowledge, only few studies involving the effect of the operative conditions of the melt-compounding process (such as the temperature profile or screw rotation speed) on the final morphology of PE blends can be found [8,16,17,18,19,20,21]. Especially, few studies dealing with HDPE/HDPE binary blends exist [8,16,20,21], although these systems are of particular interest when approaching the self-reinforcing composite field, given the possibility of obtaining peculiar shish-kebab crystalline structures, formed via structuring processing [22,23,24,25,26]. In fact, an increasing interest in the effect of unimodal, bimodal or trimodal molecular weight distributions on the final microstructure can be observed [25,26,27,28]. As an example, a lower crystallization rate and higher overall crystallization was observed for a bimodal MWD (molecular weight distribution) in comparison to a unimodal MWD [27]. In general, it has been demonstrated that having a higher degree of freedom in selecting the MW (molecular weight) values inside the PE blends allows for the proper tuning of the final properties of the material, along with its processability [28].

In this work, two commercially available HDPEs with different MWs (and, hence, viscosities) were blended, considering different weight ratios through a melt compounding step performed in a twin-screw extruder. The processing was carried out by selecting two different values of the temperature profile and screw rotation speed. More specifically, the materials were processed following a Design of Experiment (DoE) approach (2^3^ full factorial design) [29]. The obtained blends were then characterized from a thermal and rheological point of view, aiming at relating their microstructure to the adopted processing parameters. Particularly, the results of the thermal analyses were evaluated according to the response surfaces of a multilinear regression MLR model built on the experiments that were planned using the DoE approach, while the rheological data were analyzed through a PCA (Principal Component Analysis) multivariate approach [30]. Finally, the miscibility of the two HDPEs was discussed while considering three different rheological models.

## 2. Materials and Methods

### 2.1. Materials

The following commercially available HDPEs with different molecular weights (MWs) were used:

- Lupolen 5021 DX from LyondellBasell (Houston, TX, USA), selected as a high-MW polymer and hereinafter named HMW (melt flow rate (190 °C/2.16 kg) = 0.25 g/10 min; density = 0.950 g/cm^3^);
- Eraclene MP90U from Versalis (San Donato Milanese, Italy), used as a low-MW polymer and hereinafter named LMW (melt flow rate (190 °C/2.16 kg) = 7 g/10 min; density = 0.960 g/cm^3^).

### 2.2. Processing

The melt compounding was performed using a Process 11 (Thermo Fisher Scientific, Waltham, MA, USA) twin-screw extruder. The flow rate was maintained at 270 g/h, while two different temperature profiles and screw speeds were selected. In particular, two flat temperature profiles at 175 and 190 °C were used, while the screw rotation speed was maintained at 150 or 400 rpm. The processing conditions and the material composition for each extrusion were selected through a DoE approach, as explained in Section 2.4.1. The compounded materials were then rapidly cooled in a water tank and pelletized.

The formulated blends were named considering the composition and the processing conditions adopted for the melt compounding; as an example, “70HMW_175C_400” refers to the blend containing 70 wt% of HMW and 30 wt% of LMW, compounded at 175 °C and at 400 rpm.

Specimens for the rheological characterization were obtained through a compression molding step, using a hot-plate press operating at 100 bar, 190 °C, for 3 min.

### 2.3. Characterization

Differential Scanning Calorimetry (DSC) measurements were performed using a Q20 apparatus (TA Instrument, New Castle, DE, USA) on 7 ± 1 mg samples placed in closed aluminum pans. The materials were subjected to two heating ramps from 0 to 200 °C, with a heating rate of 10 °C/min, separated by a cooling ramp from 200 to 0 °C with a cooling rate of 10 °C/min. The melting temperature was obtained as the temperature corresponding to the maximum of the endothermic peak, and the melting enthalpy was obtained as the integral of the corresponding peak.

Furthermore, considering the effect that the processing parameters had already had on the pristine HMW and LMW, the establishment of a reference system was required in order to effectively compare the effects of the parameters. Specifically, the melting enthalpy of each blend was expressed referring to the second heating melting enthalpy of the commercial HMW and LMW pellets. This means that the enthalpies of the as-received pellets of HMW and LMW were firstly obtained. Then, the expected enthalpies for the blends were calculated according to Equation (1) (the values of the melting enthalpies for all the investigated materials are reported in Table S1):

(1) $$∆H_{calc}=m*∆H_{HMW}+(1-m)*∆H_{LMW}$$

where Δ*H*_calc_ is the calculated melting enthalpy, m is the concentration of HMW in the blend, Δ*H*_HMW_ is the melting enthalpy of the HMW pellet, and Δ*H*_LMW_ is the melting enthalpy of the LMW pellet.

Subsequently, the relative melting enthalpy displacement was obtained according to Equation (2):

(2) $$∆H_{\mathrm{Gap}}=\frac{∆H_{\mathrm{Exper}}-∆H_{\mathrm{calc}}}{∆H_{\mathrm{Exper}}}*100$$

where Δ*H*_Gap_ refers to the difference between the experimental and the calculated melting enthalpy, Δ*H*_Exper_ is the experimental melting enthalpy recorded during the first heating cycle of the compounded material (see values reported in Table S2), and Δ*H*_calc_ is the melting enthalpy calculated according to Equation (1). 

Considering Equation (2), the value of Δ*H*_Gap_ will be positive if the experimental enthalpy (and thus, the crystallinity) is greater than the calculated value. On the other hand, if the experimental value is smaller than the calculated one, the final value will be negative. Lastly, if the two values match, Δ*H*_Gap_ will be equal to zero. The interpretation of the data was performed using the DoE approach (please see Section 2.4.1).

The crystallinity of the samples was calculated according to Equation (3):

(3) $$\mathrm{Crystallinity}\left(\%\right)=\frac{∆H}{∆H^{0}}*100$$

considering 290 J/g [8] as the heat of fusion for a 100% crystalline PE sample ($∆H^{0}$).

Rheological tests were performed using an ARES (TA Instrument, New Castle, DE, USA) strain-controlled rheometer. The established geometry refers to a parallel plate geometry, with a diameter of 25 mm and an imposed gap of 1 mm during the test. The frequency sweep tests were performed at a strain amplitude of 10% (at which the preliminary strain sweep tests proved to be within the linear viscoelastic range for all samples), with a frequency ranging from 100 to 0.1 rad/s. The measurements were performed on each sample at two different temperatures, namely, 175 and 190 °C, in order to investigate the possible evolution of the microstructure that was achieved during processing at different temperatures. The rheological results were analyzed with a multivariate approach using PCA, as detailed in Section 2.4.2. 

### 2.4. Data Analysis

#### 2.4.1. Design of Experiments (DoEs)

To efficiently evaluate the effects of each factor that is possibly affecting the morphology of the formulated blends, an experimental design was set up. By defining fixed levels for each factor, a set of combinations is obtained, each combination corresponding to an experiment to be performed. This is the typical approach of the Design of Experiment method [29]. Firstly, the ranges of the values of interest for the three factors were considered, leading to the definition of specific values (or levels) to be used to define the factors’ combinations. The levels usually corresponded to the minimum and maximum values of the range of interest, followed by additional levels within the interval, chosen so that subsequent levels are equidistant.

Starting from the polymer’s relative concentrations, five levels of decreasing percentages of the HMW were defined for this factor: 100%, 70%, 50%, 30% and 0% (or the complementary percentages considering the concentrations of the LMW). Then, two levels were defined for both the compounding temperature factor (namely, 175 and 190 °C) and the screw rotation speed factor (150 and 400 rpm). Thus, this DoE is characterized by two factors with two levels each and one factor with five levels. The number of compounding processes performed is therefore equal to twenty (2 × 2 × 5 = 20).

Additionally, the addition of a further factor was required specifically for the rheological analyses. In this case, the analysis temperature was considered a factor with two levels (175 and 190 °C). Table 1 lists the factors and their relative levels. In the present study, the DoE will be discussed specifically for the DSC analysis.

The ranges defined for each factor define the so-called experimental domain. This domain contains virtually all the experimental conditions that can be explored and modeled. By systematically varying all the factors across their levels, the defined set of combinations homogeneously and geometrically spans the experimental domain, allowing for modeling of the behavior of a specific response across the domain itself. This means that by using these carefully selected experimental points, an estimation of the response can also be obtained in the points of the experimental domain for which no experimental value was measured.

Modeling the domain requires defining a mathematical function to describe the experimental data that were acquired according to the DoE scheme, and this can be achieved in various ways. The most common but at the same time very flexible method used in DoE is based on multilinear regression (MLR, [31]). The flexibility of MLR comes from the fact that, starting from the factors that are under examination and their defined levels, different additive terms can be included in the model’s equation, each one describing an effect that each factor can have on the response. For this reason, defining the experimental domain (factors and their levels) is strictly connected to the postulated MLR model, i.e., which terms should be included in the model’s equation.

In our case, two factors with two levels were studied, as was one with more than two levels. With two levels, only the linear terms and the interaction terms of these factors can be modeled. Over two levels, the quadratic terms can also be included, as at least one level between the minimum and the maximum values is required (the central value, if possible). Therefore, the postulated MLR model of our DoE for modeling the response (*y*) is described by Equation (4):

(4) $$y=b_{0}+b_{p_{wt}}\cdot X_{p_{wt}}+b_{T}\cdot X_{T}+b_{rpm}\cdot X_{rpm}+b_{p\_wt,T}\cdot X_{p\_wt}\cdot X_{T}+b_{p\_wt,rpm}\cdot X_{p\_wt}\;\cdot X_{rpm}+b_{T,rpm}\cdot X_{T}\cdot X_{rpm}+b_{p_{wt}^{2}}\cdot X_{p\_wt}^{2}$$

which includes three linear terms ($b_{pwt}$, $b_{T}$, $b_{rpm}$), three interaction terms ($b_{p\_wt,T}$, $b_{p\_wt,rpm}$, $b_{T,rpm}$) and one quadratic term ($b_{p_{wt}^{2}}$). Being a regression equation, the b terms represent the regression coefficients, and their interpretation based on their values and significances allow us to deduce the actual effect of each factor on the response y.

All DoE modeling using MLR was performed with the open-access software Chemometric Agile Tool [32].

#### 2.4.2. Principal Component Analysis (PCA)

Principal Component Analysis [33] is an exploratory data analysis method that is used to facilitate the interpretation of multivariate data. In fact, it reduces the data dimensions and removes the noise owing to the projection of the data onto a space of fewer dimensions. In particular, this space is defined by the so-called Principal Components (PCs), which are linear combinations of the original variables. Each PC describes a portion of the information that is contained in the modeled data, and the PCs are ordered by decreasing the amount of explained variance. Thus, by properly selecting the number of PCs, it is possible to model the actual information of the data and exclude the noise [30]. 

In the present study, PCA is applied to the rheology data with the aim of inspecting whether the screw speed, the temperature profile, the blend composition and the temperature of the rheological measurement have any effects on the rheological curves. In PCA, the factors’ levels (as discussed in Section 2.4.1) can be used as “class information”, i.e., the PCA results can be colored according to the different levels to spot possible groupings and tendencies in the so-called score plots. These scatter plots, in which pairs of PCs are plotted against the other, are one of the two main outputs of PCA, and each point on a score plot corresponds to one sample (i.e., one individual experimental rheological run). This means that samples that are close to each other will share similar features, while distant ones will have different results.

To the best of the authors’ knowledge, the application of PCA analysis to the study of the rheology of polymer blends is an innovative approach. In fact, only few works concerning PCA analysis of rheological data of asphalts [34] or bitumen [35], aqueous dispersions for cosmetic use [36], wheat-based doughs [37,38] and drug-delivering polymer systems [39] are available in the literature. Nevertheless, in all these studies, a PCA approach is applied to experimentally measured parameters (such as a cross-over modulus, zero-shear viscosity or phase angle), while in this work, the overall dependency of the complex viscosity on the frequency will be analyzed. 

In this study, the PCA toolbox for MATLAB [30], developed by the Milano Chemometrics and QSAR Research Group, was used. The toolbox is freely downloadable from the group’s website [40].

## 3. Results and Discussion

### 3.1. Rheological Behavior

**Figure 1:**
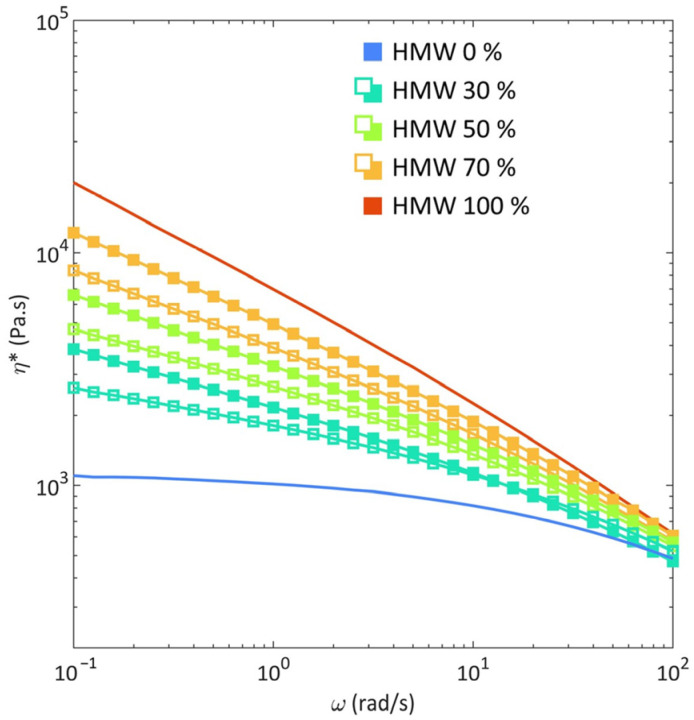
Complex viscosity (η*) as a function of the frequency (ω) for HMW (high molecular weight) and LMW (low molecular weight) (continuous lines) and blends (solid symbols), processed at 190 °C, 400 rpm, and analyzed at 175 °C. The values calculated through the logarithmic additive rule (hollow symbols) are also reported.

Figure 1 presents the complex viscosity curves for HMW, LMW and their blends processed at 190 °C and 400 rpm. As expected, the rheological behaviors of the two pristine polymers strongly differ, according to their different molecular weights. In particular, LMW shows a pronounced Newtonian behavior, with a Newtonian plateau developing in the low–intermediate frequency range and mild shear thinning in the high-frequency region. Otherwise, HMW exhibits a shear-thinning behavior throughout the whole investigated frequency interval, which is likely due to the high molecular weight of the samples, implying the formation of a dense network of entanglements which hinders the full relaxation of the macromolecular chains in the tested time interval [41]. The blends exhibit a rheological behavior that is intermediate between those of the two HDPE samples, with complex viscosity values accounting for the relative content of HMW and LMW. Nevertheless, it should be noticed that the low-frequency behavior of all the investigated blends is strongly affected by the presence of HMW; in fact, regardless of the content of the high-molecular-weight HDPE, all the blends exhibit a prominent non-Newtonian behavior at the lowest investigated frequencies. 

Aiming at evaluating the miscibility of HMW and LMW in their molten state, the experimental data were compared with the trends of the complex viscosities, obtained considering three different additive rules. In particular, the logarithmic rule (Equation (5)) and the linear rule (Equation (6)) predicting the behavior of miscible blends [42] were used, as was the diluted emulsion of the Newtonian liquid model (Equation (7)) [43]:

(5) $$\ln\eta_{B}=W_{HMW}*\ln\eta_{HMW}+{(1-W}_{HMW})*\ln\eta_{LMW}$$

(6) $$\eta_{B}=W_{HMW}*\eta_{HMW}+{(1-W}_{HMW})*\eta_{LMW}$$

(7) $$\eta_{B}=\eta_{m}*\left(1+\frac{5\varphi +2}{2\varphi +2}*n\right)$$

where *η*_B_ is the viscosity of the blend, W_HMW_ is the weight fraction of HMW, *η*_HMW_ and *η*_LMW_ are the viscosities of HMW and LMW, respectively, *η*_m_ is the viscosity of the matrix, φ is the viscosity ratio between the dispersed phase and the matrix, and n is the volume fraction of the dispersed phase. In all cases, the viscosity values of the matrices refer to LMW and HMW being compounded and analyzed in the same conditions as those of the blend.

From the comparison of the experimental data with the calculated values, it emerged that, irrespectively of the blend’s composition and of the adopted processing conditions, the diluted emulsion model is the worst-fitting one, as it does not predict reliable values, neither in the low-frequency or in the high-frequency region. On the other hand, the linear and logarithmic models perform better. In particular, both models accurately predict the rheological behavior in the shear-thinning region, especially for frequencies above 10 rad/s, while they overestimate the experimental values in the low-frequency region (as is observable in Figure 1 for the blends compounded at 190 °C, 400 rpm, and analyzed at 190 °C and in Figures S1 and S2 for all the studied materials). 

In order to gain further insights into the miscibility of the two matrices in the molten state, the Cole–Cole plots of all the investigated materials were analyzed. In fact, through this representation, the relaxation behavior of the blends can be assessed, allowing us to obtain important information about the miscibility of the polymers. In particular, the shape and smoothness of the plot of the imaginary part of the viscosity (*η*″) versus the real part (*η*′) is evaluated [9,44,45,46]. According to the literature, homogeneous polymeric materials with a single-phase microstructure are characterized by a smooth and semicircular arc, indicating the presence of a single dynamic population relaxing in a single time interval. Conversely, more complex shapes (involving the appearance of a second arc or of a linear tail) are expected for systems presenting distinct relaxation times resulting from the presence of different phases. 

**Figure 2:**
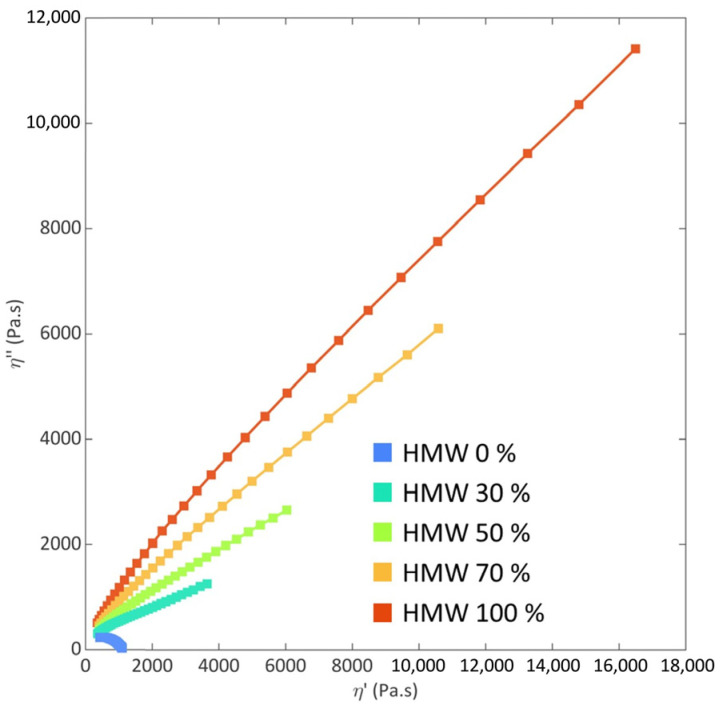
Cole–Cole plot for HMW (high molecular weight), LMW (low molecular weight) and blends, compounded at 190 °C, 400 rpm, and tested at 175 °C.

The representative Cole–Cole plots of the blend processed at 190 °C and 400 rpm (whose complex viscosity curves are presented in Figure 1) are reported in Figure 2 (the curves for all the investigated blends are plotted in Figures S3 and S4). Firstly, as already discussed for the complex viscosity, the two base HDPEs show a very dissimilar behavior, according to their different molecular weights. More specifically, the plot for LMW has a semicircular shape, indicating the complete relaxation of the macromolecules of this sample in the tested time interval. In contrast, the high molecular weight of the HMW macromolecules involves the obtainment of longer relaxation times compared to LMW, and the polymer is not able to fully relax in the same time domain. Once again, it can be observed that the behavior of the blends is intermediate between those of the two matrices. In all cases, the curves are smooth and do not show deviations from the full arc shape, indicating the presence of a unique relaxation mechanism. This result indicates the achievement of a uniform and homogeneous morphology in the molten state for all the explored HMW/LMW compositions. As already inferred from the analysis of the complex viscosity curves, the presence of HMW strongly influences the relaxation dynamics of the blends; in fact, the blend containing the lowest amount of HMW (i.e., 30 wt%) also shows a significantly higher relaxation time compared to LMW. 

**Figure 3:**
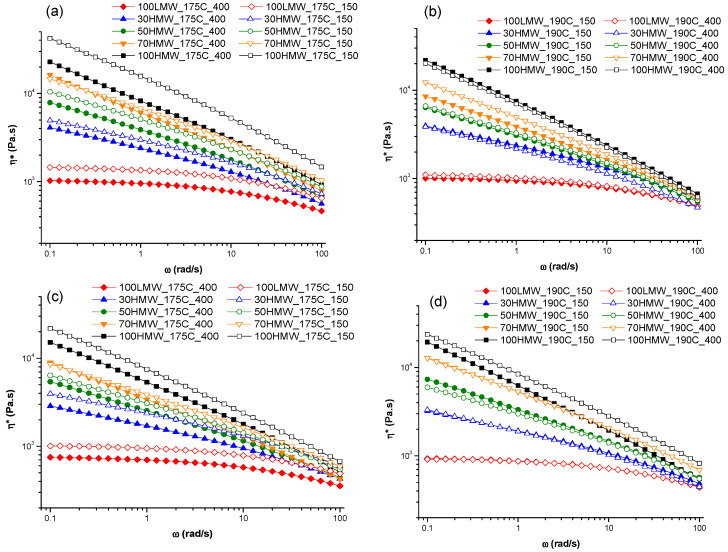
Comparison of the complex viscosity curves depending on the variation in the screw rotation speed, with a constant processing temperature and analysis temperature. (**a**) Processing temperature = 175 °C and analysis temperature = 175 °C; (**b**) processing temperature = 190 °C and analysis temperature = 175 °C; (**c**) processing temperature = 175 °C and analysis temperature = 190 °C; (**d**) processing temperature = 190 °C and analysis temperature = 190 °C.

Finally, the effects of the other three parameters (screw rotation speed, processing temperature and analysis temperature) were investigated. Figure 3 presents the comparison of the complex viscosity curves for the blends compounded at different screw speeds, maintaining a constant processing temperature and analysis temperature. The graphs reporting the comparison of the other parameters are reported in Figures S5 and S6. Firstly, from a general point of view, when considering the materials that were compounded under the same screw speed and processing temperature conditions, an amplification of the non-Newtonian behavior (i.e., a decrease in the Newtonian behavior and intensification of the shear thinning) from increasing the content of HMW can be observed. However, some differences emerge for the behavior of the 70HMW and 50HMW blends processed at 150 rpm and analyzed at 175 °C: both systems show a more pronounced shear thinning when compounded at 190 °C. 

As far as the screw rotation speed is concerned (Figure 3), the effect of this parameter is almost negligible for the blends processed at 175 °C. In contrast, for the systems compounded at 190 °C, higher values of the complex viscosity were obtained at a low screw speed. 

Additionally, the processing temperature seems to only have an effect in a few cases. More specifically, for the blend compounded at 150 rpm and analyzed at 175 °C, the blends compounded at a processing temperature of 175 °C showed a higher viscosity than the one compounded at 190 °C. Also, HMW and 70HMW compounded at 400 rpm and analyzed at 190 °C showed a higher viscosity when compounded at 190 °C. The same was the case for the 70HMW compounded at 150 rpm and analyzed at 190 °C.

### 3.2. PCA Analysis

**Figure 4:**
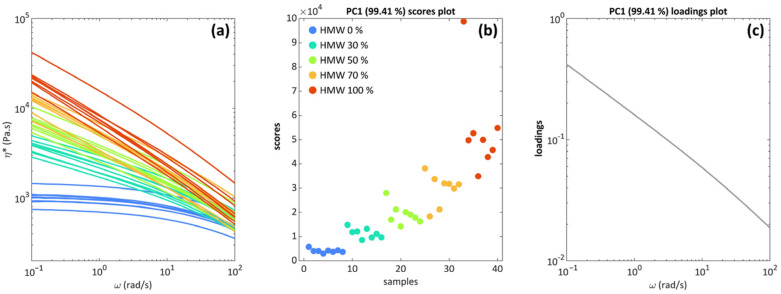
(**a**) Viscosity curves (raw data), colored according to polymer concentrations; (**b**) PCA (Principal Component Analysis) score plot of PC1, colored according to polymer concentrations (“samples” refers to all blends compounded in different conditions); (**c**) PCA (Principal Component Analysis) loadings of PC1.

To investigate the effects of the processing parameters on the rheological behavior of the blends more deeply, the results from the rheological characterization were analyzed using PCA. As a first step, PCA requires the selection of the number of PCs to be modeled [30] or, in other words, to define the dimension of the model. In our case, the selection was made on the basis of the variance that is explained by each component, but also considering the information that is displayed by each component. Some clear trends were found in PC1, while the information related to PC2 and PC3 (the next PCs of interest) were deemed too weak to be interpreted clearly. Furthermore, PC1 describes 99.56% of the total variance, leaving PC2 (0.43%) and PC3 (0.01%) with just the crumbs. So, only the information that is described by PC1 was inspected and will be commented on in the following. The information about the polymer concentrations was used to color the PCA scores that are depicted in Figure 4b. Additionally, Figure 4a presents the viscosity curves for all the samples obtained with different compositions (0, 30, 50, 70, and 100 wt% of HMW), extrusion temperatures (175 or 190 °C), screw rotation speeds (150 or 400 rpm) and testing temperatures (175 or 190 °C). It is important to highlight that, for the curves presented in Figure 4a, each color refers to the samples containing the same amount of HMW (irrespectively of the other considered parameters).

The most significant results are related to the blend concentration, and this can be clearly seen in the case of PC1, presented in Figure 4b. As the percentage of HMW increases, the differences between the blends become larger, along with the internal variability of each blend: the pure LMW (0% HMW) samples appear to be much more similar to each other than the 70% and 100% HMW blends. This difference can be noticed because with an increasing concentration of HMW, the blends become much more vertically scattered in Figure 4b: the experiments become “less reproducible”, so the factors that were varied in the DoE scheme have an enhanced influence on the blend’s properties, as will be discussed below.

The interpretation of the differences between the blends is carried out by inspecting the loading plot in Figure 4c. All the experiments consisted of a curve with decreasing values of complex viscosity as the frequency increases, which is expected considering the pseudo-plastic rheological behavior of the investigated materials. The blends appear to be mostly distinguished in the low-frequency region, which is a confirmation of the fact that the value of the zero-shear viscosity and the low-frequency behavior are crucial in determining the blends’ properties. The fact that only one PC is able to describe basically all the information that is present in the data (over 99.4% of the total variance) confirms that also from a multivariate and holistic point of view, the curves under examination follow the same viscosity changes that are described by the loadings of Figure 4c. It is very important to consider that this approach (PCA modeling of the data) did not require postulating an a priori model, so what is described in Figure 4 is the actual information that is contained in the data, but represented in a clearer way, especially regarding the within-blend variability (Figure 4b). Therefore, PCA and similar multivariate approaches, based on mathematical decomposition, allow us to retrieve the relative and absolute measurements of the differences between samples directly from the data.

The distribution depicted in Figure 4b relating to the HMW content is in accordance with the expected behavior of the viscosity when considering polymers with different MWs [8]. Moreover, the fact that the viscosities of the blends are always located between the one of LMW and that of HMW and progressively increase with the HMW content is further proof of the miscibility of the two HDPEs [47].

### 3.3. DSC Characterization

**Figure 5:**
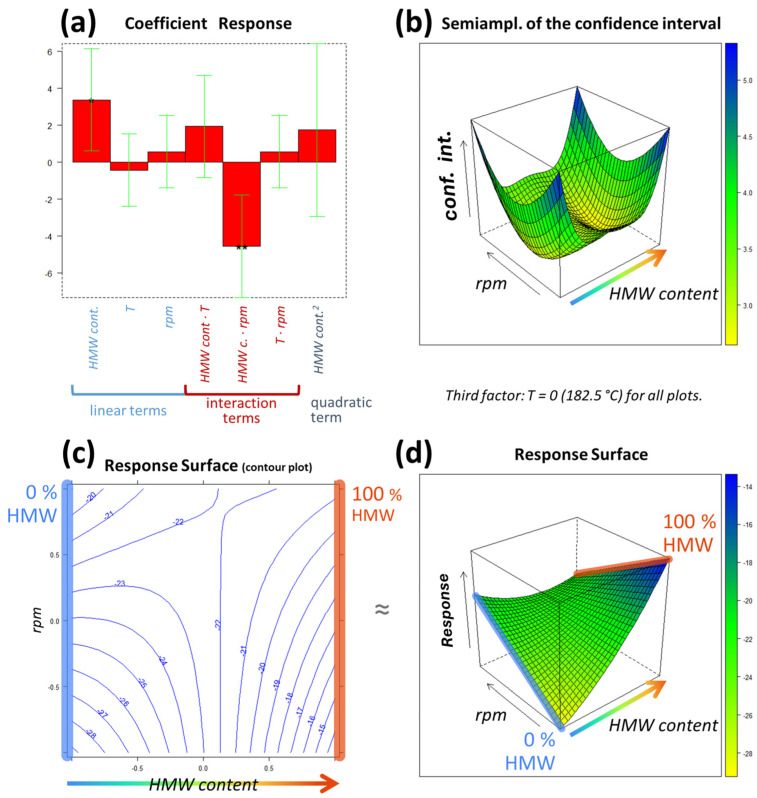
The results of modeling the DoE (Design of Experiments) with MLR (multilinear regression). The experimental domain portion inspected in the figures corresponds to the only factors for which the coefficients resulted in significant results (*HMW content* and *rpm*), while the remaining one (T) was set to its central level (T = 182.5 °C). The regression coefficients are represented in (**a**), and the response surface is depicted in two dimensions ((**c**) a contour plot) and three dimensions (**d**). The confidence interval values corresponding to the response surface are reported in (**b**).

Multilinear regression was applied to model the DoE experimental results, in which the displacement (Δ*H*_Gap_) between the experimental (Δ*H*_Exper_) and the calculated (Δ*H*_Calc_) melting enthalpy was determined for each performed experiment and thus used as the response variable *y*. According to the postulated model described in Equation (3), three linear terms (one for each factor), three interaction terms and one quadratic term were included. The resulting coefficients are visually depicted in Figure 5a, together with their confidence interval and significance. Only two terms resulted in relevant (based on the coefficient’s value) and significant (please note the asterisks in Figure 5a, which correspond to different significance levels) results: the linear term of the concentration of HMW (*HMW content*) and the interaction term between the concentration and the screw rotation speed (*HMW content* ∙ *rpm*). All other terms resulted in non-statistically significant results, and their relevance was also significantly reduced compared to the abovementioned interesting terms. For these reasons, only *HMW content* and *rpm* will be discussed. The experimental domain portion represented in Figure 5 corresponds to these two factors. 

Starting from *HMW content*, the positive value of its linear term indicates that, from a general point of view, the response increases as the percentage of HMW increases (or as the content of LMW decreases). Thus, taking Equation (2) into consideration, this means that, in general, the greater the quantity of HMW is, the closer the experimental enthalpy is to the calculated one. However, the interaction with *rpm* is also relevant, and this causes a distortion of the response surface across the experimental domain (Figure 5d), so the interpretation of the linear term of *HMW content* alone can be misleading.

To interpret these two effects together, an inspection of the response surface reported in Figure 5d is required. As can be observed, there are two extreme situations between which the surface develops: 0% HMW (pure LMW, in blue) on the left and 100% HMW on the right (in red). The effect of the interaction between the blends (*HMW content*) and the screw rotation speed (*rpm*) can be clearly seen at these two extremes. The maximum and minimum response values that are obtained within the experimental domain can be found at low *rpm* (level of −1 = 150 rpm), with the maximum response at 100% HMW (level of +1 of *HMW content*) and the minimum response at 0% HMW (level of −1 of *HMW content*). Thus, the lowest enthalpy is obtained for pure LMW processed at low rpm, while the highest enthalpy is obtained for pure HMW that is melt-compounded at low rpm. Moving to higher *rpm* values, the situation becomes practically unrelated to the content of HMW, since the response values that are obtained at the two *HMW content* extremes (0% and 100%) are essentially the same, especially considering the associated error. This effect can be more clearly observed in Figure 5c: by moving horizontally along the top part of the contour plot, the response does not vary significantly, from about −20 to about −21. Considering that the minimum error associated to the response surface values is about ±2.6 (bottom part of the color bar of Figure 5b), the difference between these two values is not significant, so they can be considered virtually equal. The visual description of the error (i.e., the confidence intervals) is provided in Figure 5b, whose dimensions correspond to the response surface of Figure 5d and the contour plot of Figure 5c: they must be interpreted jointly, as they both describe two quantities within the same portion of the experimental domain. For instance, an error of about ±5.4 is associated with both the minimum and maximum response values, which are, respectively, −29 ± 5.4 and −14 ± 5.4. This difference appears to be significant. In between the two extreme percentages (the two pure polymers) are the blends. The response surface in Figure 5d (which is the graphical representation of the MLR mathematical function) allows us to have an estimate of the response also in relation to blends that were not tested experimentally.

**Figure 6:**
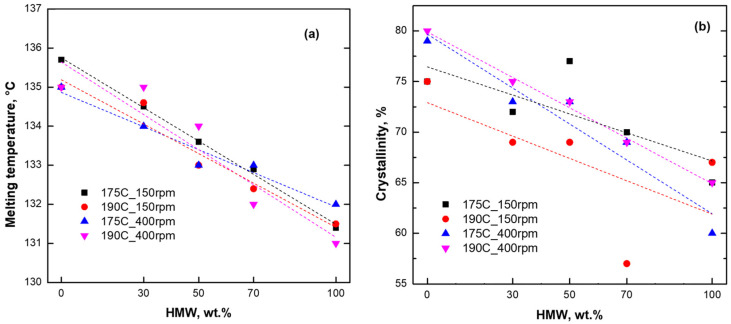
(**a**) Melting temperature and (**b**) crystallinity degree for all investigated materials as a function of HMW (high molecular weight) content. Dashed lines refer to trend of linear additivity rule.

Furthermore, DSC is a well-known indirect technique to evaluate the miscibility of blends, although there is a lack of research on HDPE/HDPE blends when considering the effect of the processing parameters [7]. In this context, Bai et al. [8] investigated the miscibility of two HDPEs with different MWs through DSC, demonstrating the presence of a single melting peak for all the blends, along with a decrease in the melting temperature with the increase in the HMW content. Also, it was observed that the crystallinity of the materials followed a linear additivity rule. In the present study, either the thermograms that were collected during the first or the second heating scan show the presence of a single endothermic peak, associable with melting phenomena (Figures S7 and S8). Additionally, important considerations are addressed regarding the second heating cycle. As is observable in Figure 6a, all the blends that were processed with the different combinations of processing parameters exhibit a decreasing trend of the melting temperature as a function of the HMW content, although the linear additivity rule applies exclusively for the materials processed at 175 °C and 150 rpm (R^2^ = 0.996). A similar behavior was noticed as far as the crystallinity of the materials is concerned. In fact, looking at the data reported in Figure 6b, the values of the crystallinity degree for the blends are intermediate (apart from 50HMW_175C_150 and 70HMW_190C_150) between those of the two starting HDPEs. Furthermore, the crystallinities of the systems processed at 190 °C and 400 rpm follow a linear additivity rule (R^2^ = 0.995), suggesting the achievement of a fully miscible blend [8]. This result can be explained when considering that the higher processing temperature, causing a decrease in the polymer viscosity, induces a more effective disentanglement of the polymer chains, promoting the achievement of a miscible blend.

Additionally, from a practical point of view, this refers to the possibility of tuning the crystallinity of the blends by selecting a specific HMW content and proper processing conditions [10].

## 4. Conclusions

The present study aimed at deepening our knowledge of the effect of the processing parameters (relative concentration, processing temperature, compounding screw speed) on the rheological and thermal behavior, as well as on the crystallinity, of homopolymer blends that were obtained through the melt compounding of two HDPEs with different molecular weights. The blends were processed according to a DoE approach, considering two levels of processing temperature and screw rotation speed and five levels of composition of the polymer blend. Then, the obtained materials were characterized using DSC and rheological measurements. The results of the thermal analysis were investigated by using the response surfaces of the MLR model, in which the displacement of the experimental melting enthalpy from the calculated one was used as the response variable. In this study, two factors were influential: the reciprocal matrix concentration and the screw speed. Furthermore, the DSC characterization indicated that high shear stresses and high processing temperatures promote the achievement of fully miscible materials. Additionally, the rheological behavior was investigated using PCA, a multivariate approach. In this case, the HMW composition was also found to be the most impacting parameter. Nevertheless, with a higher HMW content, an increase in the data dispersion in the score spaces was observed, indicating that the results are also affected by the processing temperature and the screw speed, whose effect, however, cannot be detected in the score plot. Overall, the proposed approach demonstrated that the multivariate analysis allowed us to achieve useful information about the processing/microstructure relationships in polymer-based blends, opening up new perspectives towards the application of this strategy in the study of the effect of the processing conditions on the final morphology of polymer-based complex systems, such as blended nanocomposites or hierarchically structured materials. 

## Figures and Tables

**Table 1:** Factors and levels that were defined to set up the DoE (Design of Experiment). Under the column “Code”, the short names that are used to refer to the factors discussed in the results are reported.

Factors	Code	Levels				
		−1	−0.5	0	0.5	1
Concentration of HMW (high molecular weight) (wt%)	HMW content	0	30	50	70	100
Compounding temperature (°C)	*T*	175				190
Screw rotation speed (rpm)	rpm	150				400
Rheology analysis temperature (°C)	/	175				190

## Data Availability

Data will be provided on request.

## Supplementary Materials

The following supporting information can be downloaded at https://www.mdpi.com/article/10.3390/polym16070870/s1: Figure S1. Comparison between the experimental data (obtained performing frequency sweep tests at 175 °C) and the curves obtained applying additive rules showed in Equations (5)–(7). (a) Materials processed at 175 °C, 400 rpm; (b) Materials processed at 175 °C, 150 rpm; (c) Materials processed at 190 °C, 150 rpm; (d) Materials processed at 190 °C, 400 rpm; Figure S2. Comparison between the experimental data (obtained performing frequency sweep tests at 190 °C) and the curves obtained applying additive rules showed in Equations (5)–(7). (a) Materials processed at 175 °C, 400 rpm; (b) Materials processed at 175 °C, 150 rpm; (c) Materials processed at 190 °C, 150 rpm; (d) Materials processed at 190 °C, 400 rpm; Figure S3. Cole-Cole plots for the materials analyzed at 175 °C. (a) Materials processed at 175 °C, 400 rpm; (b) Materials processed at 175 °C, 150 rpm; (c) Materials processed at 190 °C, 150 rpm; Figure S4. Cole-Cole plots for the materials analyzed at 190 °C. (a) Materials processed at 175 °C, 400 rpm; (b) Materials processed at 175 °C, 150 rpm; (c) Materials processed at 190 °C, 150 rpm; (d) Materials processed at 190 °C, 400 rpm; Figure S5. Comparison of the complex viscosity curves for the materials having different HMW content. (a) Materials processed at 175 °C, 150 rpm, test temperature 175 °C; (b) Materials processed at 190 °C, 150 rpm, test temperature 175 °C; (c) Materials processed at 175 °C, 150 rpm, test temperature 190 °C; (d) Materials processed at 190 °C, 150 rpm, test temperature 190 °C; (e) Materials processed at 175 °C, 400 rpm, test temperature 190 °C; (f) Materials processed at 190 °C, 400 rpm, test temperature 190 °C; (g) Materials processed at 175 °C, 400 rpm, test temperature 175 °C; (h) Materials processed at 190 °C, 400 rpm, test temperature 175 °C; Figure S6. Comparison of the complex viscosity curves depending on the variation of the processing temperature. (a) Materials processed at 175 °C, 150 rpm, test temperature 175 °C; (b) Materials processed at 175 °C, 400 rpm, test temperature 175 °C; (c) Materials processed at 175 °C, 150 rpm, test temperature 190 °C; (d) Materials processed at 175 °C, 400 rpm, test temperature 190 °C; Figure S7. Thermograms recorded during the first heating scans. (a) Materials processed at 175 °C, 150 rpm; (b) Materials processed at 175 °C, 400 rpm; (c) Materials processed at 190 °C, 150 rpm; (d) Materials processed at 190 °C, 400 rpm; Figure S8. Thermograms recorded during the second heating scans. (a) Materials processed at 175 °C, 150 rpm; (b) Materials processed at 175 °C, 400 rpm; (c) Materials processed at 190 °C, 150 rpm; (d) Materials processed at 190 °C, 400 rpm; Table S1: Melting enthalpy (ΔH) and crystallinity of the LMW and HMW of the pellets, recorded during the second heating cycle; melting enthalpy (ΔH) of the blends calculated according to Equation (1) and corresponding crystallinity; Table S2: Melting temperature (Tm), melting enthalpy (ΔH) and crystallinity of the investigated materials.
