# Supplementary material for: The Effect of Processing Conditions on the Microstructure of Homopolymer High-Density Polyethylene Blends: A Multivariate Approach
Source: Polymers (Basel). 2024 Mar 22;16(7):870. doi: 10.3390/polym16070870 (PMC11013753; doi:10.3390/polym16070870)
Supplement: Supplementary file 1 [file polymers-16-00870-s001.zip › polymers-2898172-supplementary.pdf]

Supporting information

The Effect of Processing Conditions on the Microstructure of Homopolymer High-Density Polyethylene Blends: A Multivariate Approach

Fulvia Cravero [1,2], Nicola Cavallini [3], Rossella Arrigo [1,2,*], Francesco Savorani [3] and Alberto Frache [1,2]

[1] Department of Applied Science and Technology, Politecnico di Torino, Viale Teresa Michel 5, 15121 Alessandria, Italy; fulvia.cravero@polito.it (F.C.); alberto.frache@polito.it (A.F.)
[2] Local INSTM Unit, 15121 Alessandria, Italy
[3] Department of Applied Science and Technology, Politecnico di Torino, Corso Duca degli Abruzzi 24, 10129 Torino, Italy; nicola.cavallini@polito.it (N.C.); francesco.savorani@polito.it (F.S.)
* Correspondence: rossella.arrigo@polito.it

Rheological behavior

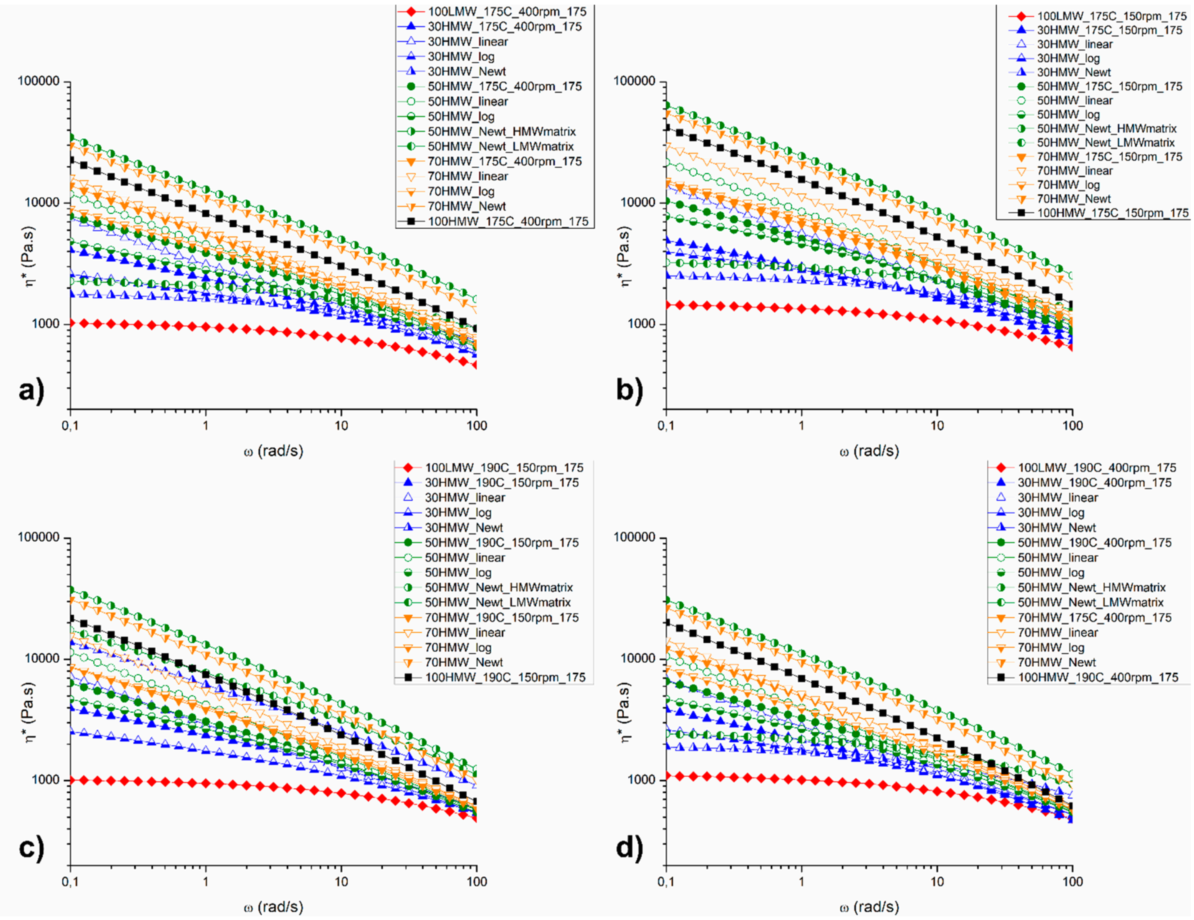

Figure S1. Comparison between the experimental data (obtained performing frequency sweep tests at 175 °C) and the curves obtained applying additive rules showed in Equations 5-7. (a) Materials processed at 175 °C, 400 rpm; (b) Materials processed at 175 °C, 150 rpm; (c) Materials processed at 190 °C, 150 rpm; (d) Materials processed at 190 °C, 400 rpm.

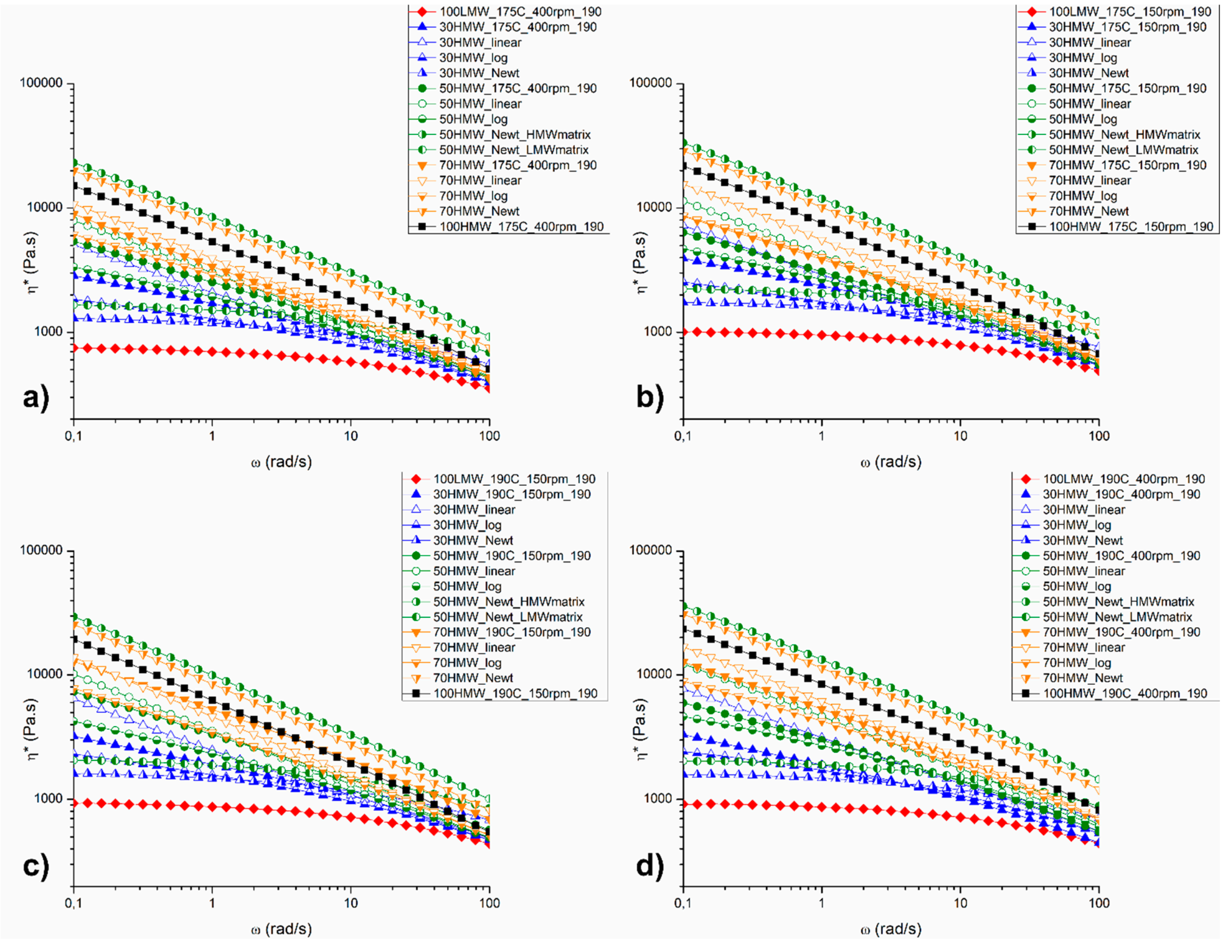

Figure S2. Comparison between the experimental data (obtained performing frequency sweep tests at 190 °C) and the curves obtained applying additive rules showed in Equations 5-7. (a) Materials processed at 175 °C, 400 rpm; (b) Materials processed at 175 °C, 150 rpm; (c) Materials processed at 190 °C, 150 rpm; (d) Materials processed at 190 °C, 400 rpm.

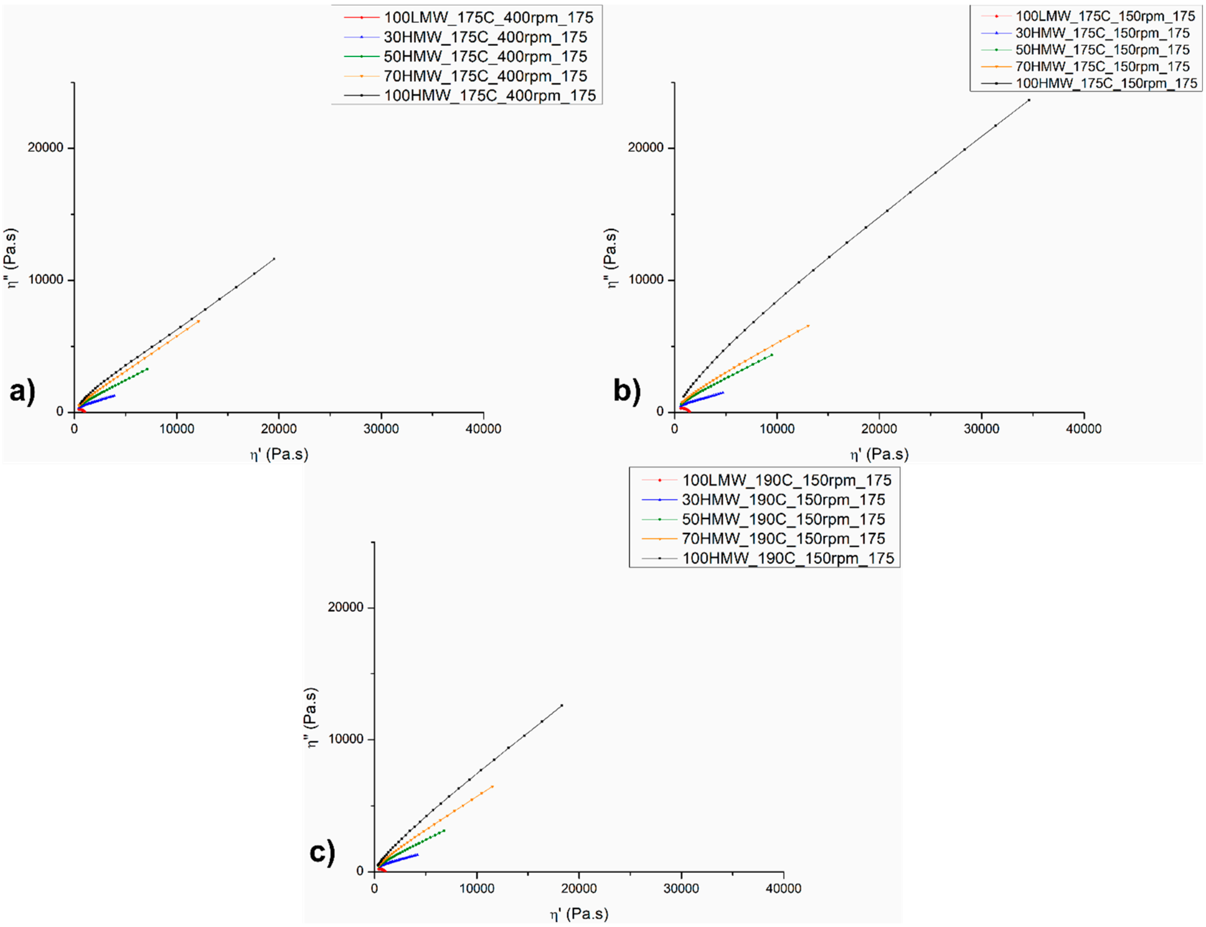

Figure S3. Cole-Cole plots for the materials analyzed at 175 °C. (a) Materials processed at 175 °C, 400 rpm; (b) Materials processed at 175 °C, 150 rpm; (c) Materials processed at 190 °C, 150 rpm.

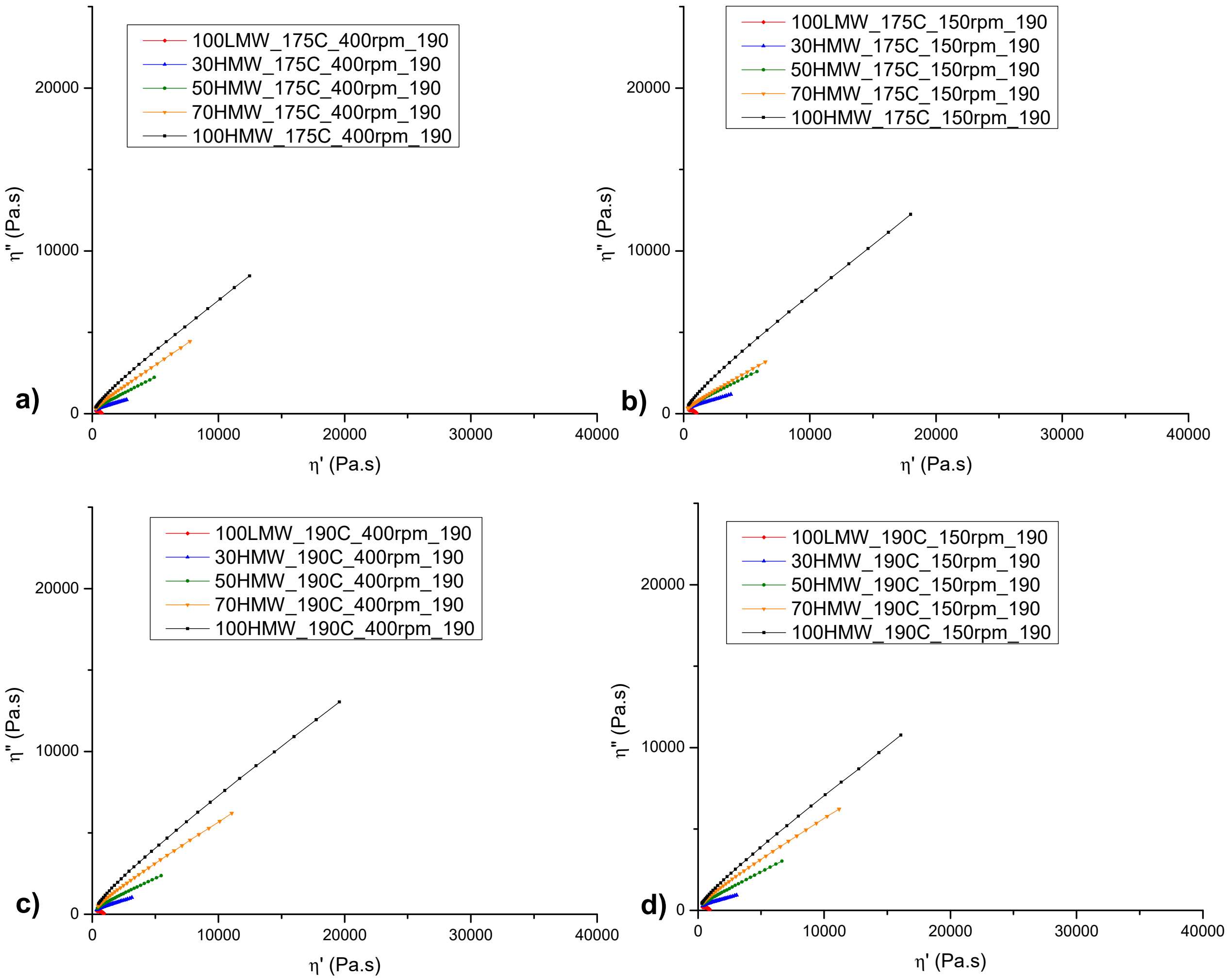

Figure S4. Cole-Cole plots for the materials analyzed at 190 °C. (a) Materials processed at 175 °C, 400 rpm; (b) Materials processed at 175 °C, 150 rpm; (c) Materials processed at 190 °C, 150 rpm; (d) Materials processed at 190 °C, 400 rpm.

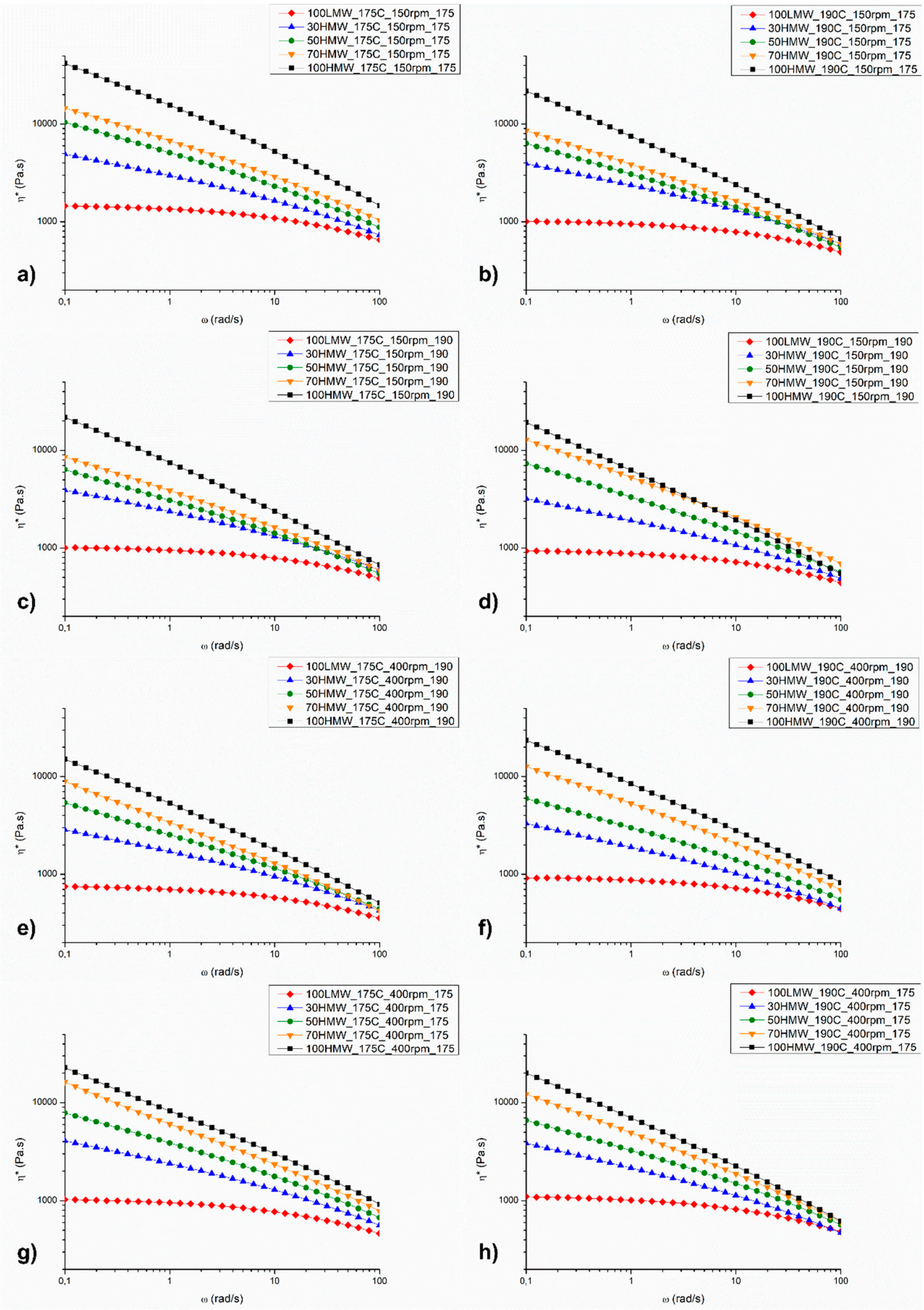

Figure S5. Comparison of the complex viscosity curves for the materials having different HMW content. (a) Materials processed at 175 °C, 150 rpm, test temperature 175 °C; (b) Materials processed at 190 °C, 150 rpm, test temperature 175 °C; (c) Materials processed at 175

°C, 150 rpm, test temperature 190 °C; (d) Materials processed at 190 °C, 150 rpm, test temperature 190 °C; (e) Materials processed at 175 °C, 400 rpm, test temperature 190 °C; (f) Materials processed at 190 °C, 400 rpm, test temperature 190 °C; (g) Materials processed at 175 °C, 400 rpm, test temperature 175 °C; (h) Materials processed at 190 °C, 400 rpm, test temperature 175 °C.

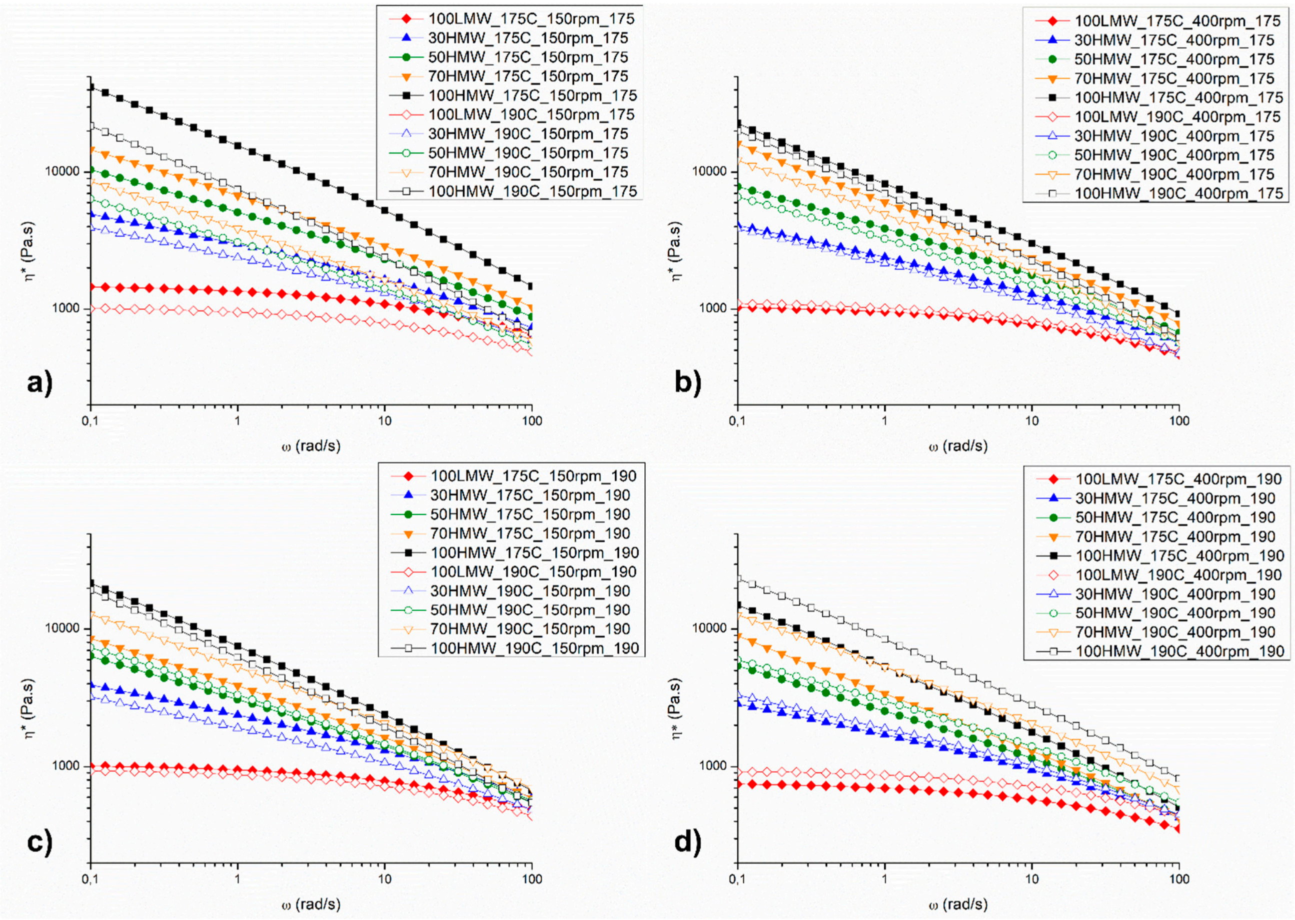

Figure S6. Comparison of the complex viscosity curves depending on the variation of the processing temperature. (a) Materials processed at 175 °C, 150 rpm, test temperature 175 °C; (b) Materials processed at 175 °C, 400 rpm, test temperature 175 °C; (c) Materials processed at 175 °C, 150 rpm, test temperature 190 °C; (d) Materials processed at 175 °C, 400 rpm, test temperature 190 °C.

<u>DSC Analyses</u>

Table S1. Melting enthalpy (ΔH) and crystallinity of the LMW and HMW of the pellets recorded during the second heating cycle; ΔH of the blends calculated according to Eq. 1 and corresponding crystallinity.

Material	DH (J/g)	Crystallinity (%)
LMW pellet 2nd heating	231	80
HMW pellet 2nd heating	187	64
30HMW calculated value	218	75
50HMW calculated value	209	72
70HMW calculated value	200	69

Table S2. Melting temperature (T_m), melting enthalpy (ΔH) and crystallinity of the investigated materials.

Material	Tm (°C)	ΔH (J/g)	Crystallinity (%)
100LMW_175C_400	139	208	72
30HMW_175C_400	136	172	59
50HMW_175C_400	136	171	59
70HMW_175C_400	135	164	57
100HMW_175C_400	133	151	52
100LMW_190C_400	136	189	65
30HMW_190C_400	137	177	61
50HMW_190C_400	136	174	60
70HMW_190C_400	134	167	58
100HMW_190C_400	131	157	54
100LMW_175C_150	136	177	61
30HMW_175C_150	135	173	60
50HMW_175C_150	135	182	63
70HMW_175C_150	134	170	59
100HMW_175C_150	132	160	55
100LMW_190C_150	136	180	62
30HMW_190C_150	136	167	58
50HMW_190C_150	134	167	58
70HMW_190C_150	133	166	57
100HMW_190C_150	132	168	58

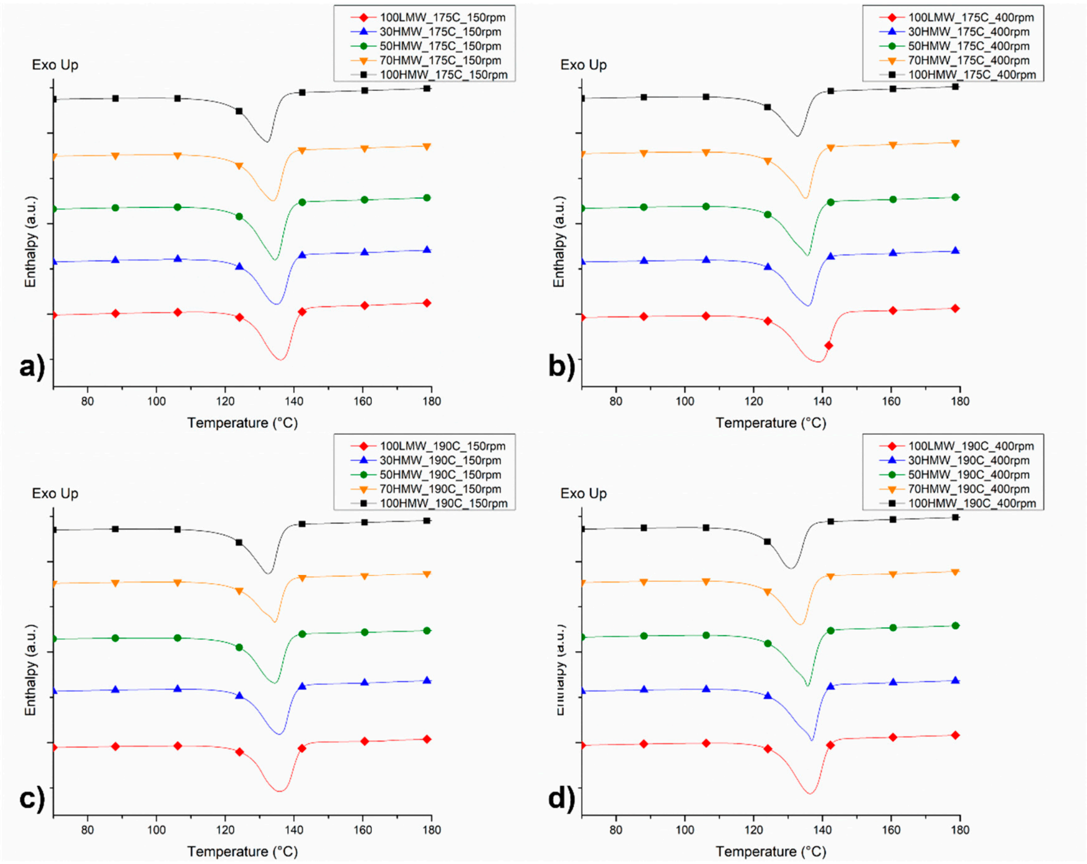

Figure S7. Thermograms recorded during the first heating scans. (a) Materials processed at 175 °C, 150 rpm; (b) Materials processed at 175 °C, 400 rpm; (c) Materials processed at 190 °C, 150 rpm; (d) Materials processed at 190 °C, 400 rpm.

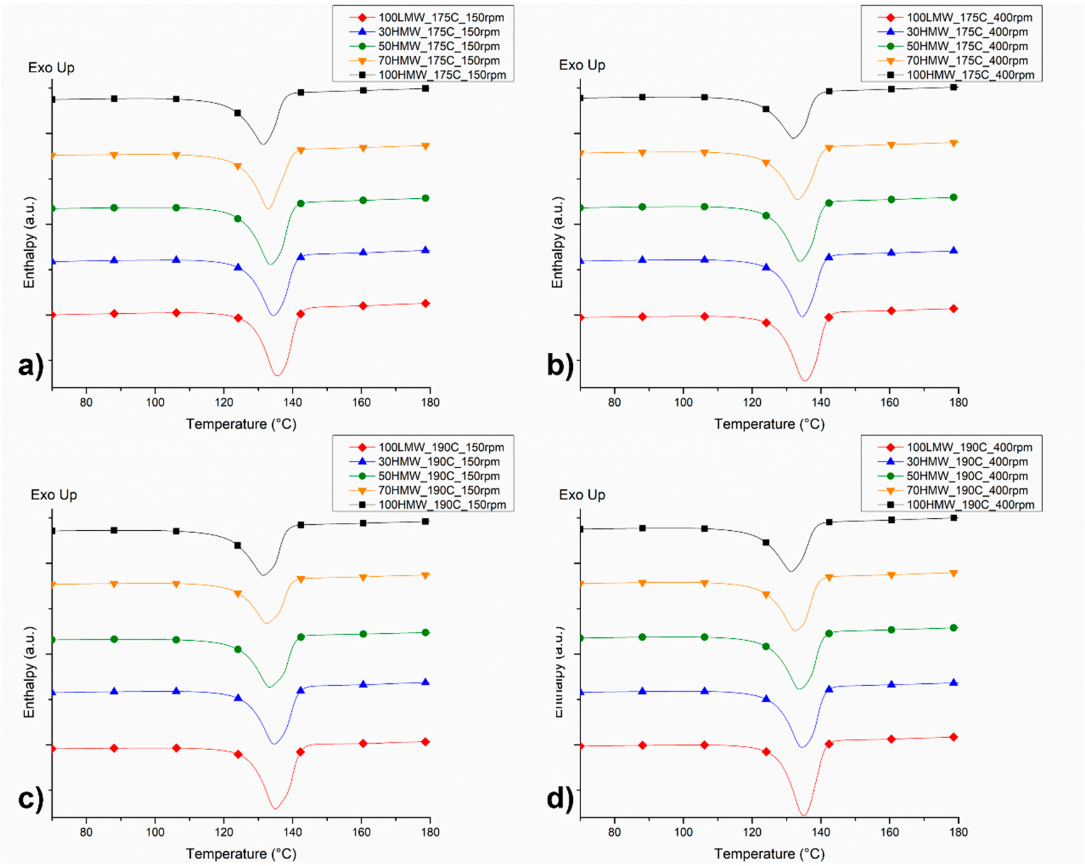

Figure S8. Thermograms recorded during the second heating scans. (a) Materials processed at 175 °C, 150 rpm; (b) Materials processed at 175 °C, 400 rpm; (c) Materials processed at 190 °C, 150 rpm; (d) Materials processed at 190 °C, 400 rpm.
